# Editorial: Youth, health and development in diverse cultures and contexts

**DOI:** 10.3389/fpsyg.2023.1247821

**Published:** 2023-07-18

**Authors:** Nora Wiium, Bin-Bin Chen, Diego Gomez-Baya, Pablo A. Pérez-Díaz, Elizabeth Trejos-Castillo, Laura Ferrer-Wreder

**Affiliations:** ^1^Department of Psychosocial Science, University of Bergen, Bergen, Norway; ^2^Department of Psychology, Fudan University, Shanghai, China; ^3^Department of Social, Developmental and Educational Psychology, Universidad de Huelva, Huelva, Spain; ^4^Institute of Psychology, Austral University of Chile, Sede Puerto Montt, Puerto Montt, Chile; ^5^Human Development and Family Sciences, Texas Tech University, Lubbock, TX, United States; ^6^Department of Psychology, Stockholm University, Stockholm, Sweden

**Keywords:** health, development, children, adolescents, emerging adults, diverse cultures and contexts

## 1. Introduction

This editorial emanates from the Research Topic entitled: “*Youth, health and development in diverse cultures and contexts*.” The Research Topic is a response to an invitation concerning the “World Day for Cultural Diversity for Dialogue and Development”, which is held every year on May 21st to celebrate not only the richness of the world's cultures, but also, the essential role of intercultural dialogue for achieving peace and sustainable development. With this mission, our motivation was to bring together a collection of studies on the health and development of youth and emerging adults, with a focus on the role of personal resources like competencies, skills, and self-perception, as well as the environmental, contextual, and relational features of social contexts. We sought to present the direct and indirect influence of personal and contextual factors with samples that reflect diverse cultures and contexts. A second motivation was to advance psychological research that has primarily featured American and other Western samples, settings, and scholars (Thalmayer et al., 2021) to include enquiries and voices from the majority world and understudied settings, by which the current collection extends contributions to our previous Research Topic on “*Positive youth development, mental health, and psychological well-being in diverse youth”* with Frontiers in Psychology.

Employing an ecological theoretical approach and perspectives from Positive Youth Development (PYD) frameworks, such as the 5Cs of PYD (Lerner et al., 2023) and the Developmental Assets Profile (Benson, 2007), the aim was to highlight how the person-environment interactions and associated developmental outcomes converge or diverge across cultures and contexts. Briefly, the 5Cs of PYD specify five positive outcomes of Competence (academic, social, vocational skills), Confidence (sense of mastery, positive identity, self-worth), Character (integrity, moral commitment, personal values), Connection (healthy relation to community, friends, family, school) and Caring (empathy and sympathy) (Geldhof et al., 2014). These are thriving indicators that emerge from adaptive interactions between youth and significant others as well as interactions with resources in different ecological settings (Lerner et al., 2023). The developmental assets are personal strengths and contextual resources that can contribute to the person-environment interaction, and consequently, to young people's health and development (Benson, 2007; Syvertsen et al., 2021).

The articles in the current collection extend the empirical evidence of the ecological theoretical approach and the PYD frameworks by presenting findings on the skills, resources, opportunities, and structures in the ecology of young people that can have significant implications for their health and development. Embracing a positive and protective approach, we depart from the domineering deficit focus on youth, while presenting a novel collection of studies on youth and emerging adults. Contributors of the articles within this collection are research partners of the Cross-National Project on Positive Youth Development (CN-PYD; Wiium and Dimitrova, 2019), who represent an international and multidisciplinary panel of experts on youth development as well as scholars from various educational institutions around the globe. By focusing on young people from diverse cultures and contexts, the collection of articles represents a holistic view of youth health and development along with the determining factors embedded within the different levels of their ecology.

The article collection adopts a comprehensive perspective, focusing on both negative (i.e., detrimental) and positive (i.e., beneficial) developmental processes in the study of health and development. Moreover, while much of the research on health and development among youth and emerging adults has emanated from the U.S. and other Western countries, contributions to our collection cover a broader geographical scope and setting by featuring research studies and researchers from several understudied non-Western contexts. Thus, our collection provides new insights into the health and developmental processes of youth and emerging adults from the majority population.

The Research Topic includes 19 articles on children, adolescents, and emerging adults from Australia and many different countries in Asia, Europe, and South America, with corresponding researchers from these continents. Comprising studies from both majority and minority populations, the collection provides a richer perspective of the conditions of youth and emerging adults as well as an inclusive voice of researchers from the global majority population. The diverse settings allow for the documentation of any similarities that may exist across cultures and contexts, and the identification of the unique ways culture and context can influence health and development, thus, avoiding biases in the study of determining factors and interpretation of findings.

## 2. Highlights from the articles in the Research Topic

Varying study designs, including cross-sectional, longitudinal and intervention were applied in the 19 empirical and methodological articles. In addition, the Research Topic reflected a broad scope of themes related to mental health, thriving and wellbeing of young people, adjustment problems and vulnerability. These research themes were based on data collected before, during and after the first year of the COVID-19 pandemic. In the following, we present highlights of the 19 articles to provide insights into some of the themes and novel contributions of the Research Topic.

### 2.1. Methodological articles

Two methodological articles were included in the Research Topic. One of them by Thomas et al.'s was a cross-sectional study that tested the psychometric properties of a teacher-reported measure of young children's social emotional competence, using the Social Competence Scale—Teacher Edition (SCS-T). The comparative study was based on samples from two countries: Pakistan (*n* = 396, 49% males) and Sweden (*n* = 309, 53% males), reflecting two contexts of varied economic resources and conditions, cultural norms, and educational experiences. Children aged 4–6 years old participated in the study. In structural equation modeling, the bi-factor confirmatory factor analysis model was found to fit the data well for both countries. Invariance testing showed that most of the items were not directly comparable across these samples, and the findings make clear the need for further instrument development that is culturally relevant and rooted.

In the second methodological article, Gull et al. conducted two studies, investigating the reliability and validity of virtual systematic social observation (virtual SSO) using Google Street View in a Swedish neighborhood context. In study 1, which focused on interrater reliability and construct validity, comparing ratings conducted in-person to those done using Google Street View, the results indicated that scales for physical decay, neighborhood dangerousness, and physical disorder were reliable and had adequate interrater reliability, high consistency across methods, and high internal consistency. For results from study 2 that focused on the criterion validity of virtual SSO, the authors observed higher levels of physical decay, neighborhood dangerousness, and signs of garbage or litter in postal code areas with lower income levels compared to those with higher income levels.

### 2.2. Articles on mental health, thriving, and well-being of young people

Several articles applying different methodological designs and sample types reported on new findings in the study of positive youth developmental outcomes. For example, using a cross-sectional design, Kadir and Rusyda examined the significant role of internal and external developmental assets, creativity, and thriving on mental health in a sample of 394 Malaysian emerging adult students (*N* = 394, 67% female; 18–26 years old, *M*_*age*_ = 21.5) attending public and private universities. Findings from regression analysis indicated that participants' mental health was significantly associated with positive identity, the support they received, their own creativity, and thriving. In addition, indirect effects of positive identity and support on mental health through creativity and thriving were observed.

In another cross-sectional study involving 495 Brazilian college students (74.5% female; 18–33 years old, *M*_*age*_ = 23.36 years), de Jesus et al. examined associations of thriving indicators (the 5Cs of PYD) with social connections' perception (family, community, academic), mental health, and stressful events and observed several positive associations between the 5Cs and connection with family, community, and university. However, Caring was not significantly associated with the social connection variables. The findings also indicated that Connection, Confidence, and Competence were positively associated with positive mental health in university students. In general, the experience of stressful events was negatively related to the 5Cs, although the association with Caring was positive.

Furthermore, Nouri et al. conducted a cross-sectional study that assessed the moderating effect of psychological hardiness on the association between youth voice and the 6Cs of positive youth development (Competence, Confidence, Connection, Character, Caring, and Contribution), with program safety and engagement as mediators. Participants were 436 first-year undergraduate students between the ages of 19 and 24 years old (*M*_*age*_ = 21.19 years) from public universities in Malaysia (65.6% female). The results indicated positive associations between youth voice and indicators of positive youth development. In addition, the association was partially mediated by program engagement but not safety; and the indirect pathway through program engagement was more predictive for hardier youth.

In a study published as a brief report, Zhang et al. examined the development of visual aesthetic sensitivity across age and gender among students in China. The influence of artistic training was also assessed. Aesthetic sensitivity is the scientific terminology for Eysenck's “good taste” and reflects the individual's ability to identify aesthetic quality and judge the quality of artworks. A cross-sectional sample of 2,387 youth from 9 to 22 years of age (youth ages 16–17 excluded) participated in the study. The results indicated a relative stability in visual aesthetic sensitivity from 9 to 12 years of age and an increase at age 13. At ages 15, 19, and 20, girls had better visual aesthetic sensitivity compared to boys. In addition, artistic training was found to improve visual aesthetic sensitivity among Chinese students.

### 2.3. Contributions on risk behaviors and adjustment problems

In a third category of articles, a number of risk behaviors and adjustment problems were studied in relation to PYD attributes or participant characteristics. For instance, Xiang et al. examined the links between cyberbullying and internet gaming disorder (IGD) along with the potential role of PYD attributes in these links in cross-sectional research. Participants were 463 Chinese adolescents (246 males; aged 11 and 18 years old, *M*_*age*_ = 15.06 years). In line with the results, cyberbullying was positively associated with IGD, after controlling for gender and age. In addition, cyberbullying and IGD were found to be negatively associated with PYD attributes. PYD attributes also mediated the associations between cyberbullying and IGD.

In another study on internet gaming disorder, Gan et al. explored how positive factors in the school subsystem could effectively prevent adolescents from bullying. The authors also studied the multiple mediation effects of intentional self-regulation (ISR) and internet gaming disorder (IGD) on the association between school assets and bullying. The sample consisted of 768 Chinese adolescents (*N* = 796 at T1, 53.8% male; *M*_*age*_ = 13.91 years) who were involved in a post-pandemic two-wave design study. The results indicated that school assets at T1 were negatively related to bullying at T2. Moreover, ISR and IGD, both at T2 were found to mediate the association between school assets at T1 and bullying at T2.

Zhao et al. examined differences in sexual knowledge, attitudes, behaviors, seeking behaviors for sex-related knowledge, and sexual and reproductive health (SRH) outcomes among only-child students and students with siblings across gender and regions in China. A total of 49,569 participants (66% male) between ages 17 and 24 (*M*_*age*_ = 19.79) were involved in the cross-sectional study. From the results, only-child students compared to those with siblings reported higher sexual knowledge, more liberal sexual attitudes, and fewer risky sexual behaviors. In addition, female students and students who resided in rural areas were more likely to report seeking behaviors for sex-related information (offline and online), after socio-economic factors, parent-child relationships, and sexuality education were accounted for.

Ran et al. conducted a study among male college students in China to assess the awareness of Human Papillomavirus (HPV) infection and HPV vaccine as well as their willingness to take the vaccine. This cross-sectional study involved 912 male college students (*M*_*age*_ = 20.42). The findings revealed that only 24.34% of participants had a “good knowledge” of HPV and the vaccine, and that 34.54% of the sample showed a “positive attitude” toward the vaccine after being informed of HPV and the vaccine. Immune persistence, side effects, pricing strategy, and participants' self-assessment of HPV infection were the main factors found to be influencing HPV vaccination.

In another study involving participants living in China, Liu et al. examined the role of socioeconomic status in different trajectories of depressive symptoms. The sample comprised 652 Chinese college freshmen (64.9% female; *M*_*age*_ = 18.6) who were followed four times across 4 months. Findings from Latent Growth Mixture Model revealed three trajectories of depressive symptoms: normal group (73.1%), depression risk group (20.7%), and depression deterioration group (6.1%). Furthermore, in multinomial logical regression, the findings indicated that for the normal group vs. depression deterioration group comparison, subjective social status significantly decreased the probability of freshmen belonging to the depression deterioration group. For the normal group vs. the depression risk group, subjective social status decreased the likelihood of belonging to the depression risk group. Age and left-behind experience also had varied influence on the trajectories of depressive symptoms found among freshmen college students.

In Kosovo, Uka et al. explored the potential role of external and internal developmental assets in treating clients with depression and anxiety disorders using Internal Cohesion Psychotherapy (ICP). The participants were ten young Kosovars (nine females and one male; *M*_*age*_ = 26.10) who participated in at least five sessions of ICP. In-depth semi-structured interviews were conducted to gather information about clients' experiences with ICP and the presence of developmental assets. The results revealed that ICP effectively treated depression and anxiety, while clients acknowledged the significant role of developmental assets in their psychotherapy sessions.

### 2.4. Empirical evidence on health, wellbeing, and development in vulnerable times and samples

A fourth category of articles included in the Research Topic were studies that focused on vulnerable times and samples. In a Chinese sample of 112 children with hearing loss, Wang et al. explored the influence of home literacy environment on the literacy development of the children along with possible mediating effects of reading interest and parent-child relationship. The study was cross-sectional and involved children with hearing loss from China's special education schools for the deaf. The participants were from 2 to 13 years of age. For the results, home literacy environment significantly predicted better literacy development of children with hearing loss. This effect was totally mediated by more reading interest and a better parent-child relationship.

In another Chinese sample comprising vulnerable children, Zheng et al. examined the association of internal migration with depression among migrant and left-behind children. Gender as a moderator as well as the indirect role of social relationships were also assessed. Participants (*N* = 2871; *M*_*age*_ = 14.62) comprised a representative sample of students enrolled in the 8th year at school; 1,430 were migrant children and 1,441 were left-behind children. The results showed that migrant children had less depression than left-behind children. Compared to left-behind children, in migrant children, internal migration was positively associated with parent–child relationships and peer relationships, which in turn reduced their depressive symptoms. Furthermore, the observed difference in mental health between migrant children and left-behind children was more prominent for boys relative to girls. The results also indicated that migrating with parents was helpful in reducing children's depressive symptoms compared to being left behind.

In Albania Miconi et al. conducted a study on risk behaviors and wellbeing among Egyptian and Roma adolescents during the pandemic. The authors also explored available personal and contextual assets among adolescents together with associations between personal and contextual assets, risk behaviors, and wellbeing. The cross-sectional sample included 201 participants (47% girls, *M*_*age*_ = 16.63). Findings from regression analysis revealed high levels of risk behaviors during the pandemic, with boys generally reporting more risk behaviors than girls. In addition, low levels of wellbeing as well as personal and contextual assets were stated, with girls scoring higher on family assets, positive values, and social competence than boys. Positive identity was significantly associated with wellbeing.

In Slovenia, Kozina and Wiium conducted a three-wave longitudinal study to track the development of the 5Cs of positive youth development in a school year, during the COVID-19 pandemic. The sample was 1241 Slovenian youth (59.5% female; ages 13–19 years, *M*_*age*_ = 15.35) attending lower or upper secondary school. The results indicated a significant decrease in Connection, Caring, and Character, but an increase in Competence and Confidence from the beginning to the end of the school year. Moreover, gender and school level (lower vs. upper secondary) played significant roles in the longitudinal development of several of the 5Cs, while age was not significantly related to any of the pathways.

In Chile, Pérez-Díaz et al. tested the hypothesis that positive identity was the core internal developmental asset explaining psychological wellbeing and that Confidence and Character had indirect associations with this relation. The cross-sectional sample comprised 261 participants (72% female; *M*_*age*_ = 22 years old), who were invited to take part in an online survey during the pandemic. Findings from structural equation modeling indicated a good model fit, and Positive identity was found to be significantly associated with psychological wellbeing. In addition, the participants' level of Confidence and Character was indirectly associated with the Positive identity—psychological wellbeing link.

In Australia, Chmiel et al. did a cross-sectional study on creativity in lockdown, to investigate how Australians in four different age groups (18–24, 25–34, 35–54, and 55+) engaged in artistic creative activities (ACAs) to support their mental health during the 2020 pandemic lockdowns. The sample consisted of 653 participants from the public. Participants who subsequently ranked undertaken ACAs in terms of effectiveness at making them “feel better,” as well as those who had engaged in musical ACAs were also asked to complete the Musical Engagement Questionnaire (MusEQ). The results indicated that younger participants overwhelmingly rated musical activities as most effective, while those aged 55+ rated non-musical activities as most effective. These differences were further supported by ratings of all six MusEQ subscales, with the youngest participants using music in significantly different ways during the pandemic (e.g., for emotion regulation and to perform a social identity) than participants in all other age groups.

Finally, in China, Zhu et al. conducted a study that sought to promote meaning in life (MIL) and wellbeing among university students during the pandemic via a service-learning (SL) course that allowed university students to apply academic knowledge in serving the community. The intervention program was carried out through a one-group pretest-posttest design, with a sample of 229 undergraduates (*M*_*age*_ = 20.86). The students were required to spend 135-h study effort in the SL course (10 h of e-learning module, 30 h of lectures, 40 h of direct services, 37 h on service preparation and post-service integration and reflection, and 18 h of reading and self-study). The intervention led to significant positive changes in MIL and wellbeing. In addition, the results revealed that pretest MIL scores positively predicted posttest scores of wellbeing, but not vice versa. As anticipated, the authors found a close association between improvement in MIL and the positive changes in both psychological and subjective indicators of wellbeing.

## 3. Conclusions about the 19 articles

The 19 articles in the Research Topic along with the broad range of Research Topics that were addressed, highlight the significant role of factors at the different levels of youth ecology in facilitating health and development in intervention programs and other youth initiatives. The findings from both majority and minority populations strengthen this assertion. In line with the findings of the article collection, personal factors, and resources within varied youth contexts, such as home, school, neighborhood, and local community, all appear to have unique and complementary roles. Moreover, the findings also align with the theoretical assumption of the ecological model (Bronfenbrenner and Morris, 2006), and the person-context interaction proposed by the PYD theoretical framework with its foundation in the relational developmental systems perspective (Overton, 2003).

Indeed, the relational developmental perspective's (Overton, 2003) emphasis on the importance of plasticity in youth development is an optimistic view that suggests that actors and stakeholders in their interaction with youth can make meaningful changes and contributions toward their health and development with the support, opportunities, and services they provide (Pittman et al., 2003). From the findings of the article collection, the support, opportunities, and services that are offered do not only enhance health, wellbeing, and development, but they also have preventive or protective effects against risk behaviors and adjustment problems. In our Research Topic, this appears to be true for both majority and minority youth, youth samples from general populations as well as those youth developing under unique circumstances or in vulnerable situations.

## 4. Looking forward: advancing research on ecological settings

Although varied youth contexts were implicated in the study of the determining factors of young people's health and development, there was very little focus on the interaction between these contexts. The ecological theoretical assumption is that human development is driven or supported by the dynamic interaction between the individual and contexts and that this interface takes place simultaneously within and between contexts (Bronfenbrenner and Morris, 2006). More research is therefore needed to determine how professionals and other stakeholders from different youth contexts can work together with young people to provide a more comprehensive intervention that considers strategies, resources, and opportunities across contexts. Equally important are research efforts that will uncover the needs of individuals in marginal or vulnerable situations to allow for tailored interventions.

Moreover, due to globalization, modernization and technological advancements, the context of young people is widening. Today, many young people take advantage of these advancements in their search for support, opportunities, and services by visiting online spaces that can offer these resources. Indeed, while physical settings like the home, school, neighborhood, and local community will continue to be important for youth health and development, digital space is becoming the new context for youth development. Proposals have already been made to extend top ecological models, such as Bronfenbrenner's ecological theory to include digital space as a new youth context (Navarro and Tudge, 2022). This inclusion will not only provide insights into the “what” and “how” the use of digital space can influence youth health and development within and across cultures, but as well, how the physical and digital world of youth interact to determine their health and wellbeing.

## 5. Conclusions

The goal of the “World Day for Cultural Diversity for Dialogue and Development” is to observe the richness of the world's cultures, as well as the fundamental role of intercultural dialogue for achieving peace and sustainable development. To achieve this goal, all young people, both majority and minority, and including those in marginal or vulnerable conditions from all youth contexts and cultures would need to be considered in this dialogue and development. Accordingly, youth voices and choices are needed in the enquiry of determining factors, but also in the planning and implementation of policies and programs that affect their own health, wellbeing, and development. Ideally, an arena that promotes adaptive interactions between youth and stakeholders from multiple ecological settings is required for youth people to reap the full benefit of the support, opportunities and services that are provided. This will eventually contribute to the promotion of peace and sustainable development that the “World Day for Cultural Diversity for Dialogue and Development” hopes to achieve.

## Author contributions

NW and LF-W were responsible for the original draft writing. B-BC, DG-B, PP-D, and ET-C contributed to the original draft and revisions. All authors read and approved the version for publication.
